# Mature murine supraspinatus tendons demonstrate regional differences in multiscale structure, function and gene expression

**DOI:** 10.1371/journal.pone.0318809

**Published:** 2025-02-20

**Authors:** Michael S. DiStefano, Stephanie N. Weiss, Courtney A. Nuss, Rebecca L. Betts, Biao Han, Andrew F. Kuntz, Louis J. Soslowsky

**Affiliations:** 1 McKay Orthopaedic Laboratory, University of Pennsylvania, Philadelphia, Pennsylvania, United States of America; 2 Department of Bioengineering, University of Pennsylvania, Philadelphia, Pennsylvania, United States of America; University of Minnesota Medical School, UNITED STATES OF AMERICA

## Abstract

The hierarchical structure of tendon dictates its ability to effectively transmit loads from muscle to bone. Tendon- and site-specific differences in mechanical loading result in the establishment and remodeling of structure, as well as associated changes in composition throughout development and healing. Previous work has demonstrated region-specific differences in the response of collagen fibrils to mechanical loading within the insertion region and midsubstance regions of mouse supraspinatus tendons using atomic force microscopy. However, multiscale structure, function, and gene expression differences between the insertion and midsubstance of the supraspinatus tendon have not yet been linked together in a comprehensive study. Therefore, the purpose of this study was to elucidate site-specific hierarchical structure, function, and gene expression differences in mouse supraspinatus tendons. Supraspinatus tendons from day 150 wild-type C57BL/6 mice were harvested for regional mechanics, histology, transmission electron microscopy (TEM), and quantitative polymerase chain reaction (qPCR). Mechanical testing revealed that the midsubstance region demonstrated a greater modulus and increased collagen fiber realignment compared to the insertion region. Histological scoring demonstrated greater cellularity and more rounded cells in the insertion region. TEM analysis showed differences in collagen fibril diameter distributions between the two regions, with a shift towards smaller diameters observed at the insertion region. Gene expression analysis identified several genes that were differentially expressed between regions, with principal component analysis revealing distinct clustering based on region. These findings provide insight into the regional heterogeneity of the supraspinatus tendon and underscore the importance of considering these differences in the context of tendon injury and repair, contributing to a better understanding of tendon structure-function and guiding future studies aimed at elucidating the mechanisms underlying tendon pathology.

## Introduction

Tendons are dynamic biomechanical structures responsible for transmitting forces from muscles to bones, facilitating movement, and maintaining joint stability. The structural integrity and functional performance of tendons are influenced by their complex hierarchical structure, which relies on coordination of multiscale regulatory processes, such as the formation of fibrils from collagen molecules, the assembly of fibril bundles to form fibers, and the recruitment of fibers to form fascicles [1]. Tendon development and maturation, along with site-specific differences in mechanical loading, result in the establishment and remodeling of this hierarchical structure as well as associated changes in its composition. This allows different sites of the same tendon to respond efficiently to load with respect to their distinct functional requirements [2].

Tendons appear homogeneous on gross inspection, but there is substantial mechanical, structural, and compositional diversity [3]. Tendons in different anatomical locations, as well as unique regions of the same tendon, have different functional requirements that must be maintained through a complex, spatially-dependent regulation of tissue homeostasis [4]. These regional differences are crucial for optimizing tendon function in response to localized mechanical demands. For instance, the insertion region of tendons often undergoes compressive loading, leading to adaptations such as increased proteoglycan content and fibrocartilage formation [5]. In contrast, the midsubstance region of tendons primarily experiences tensile forces, promoting the alignment of collagen fibers to enhance tensile strength [6]. This is especially important in tendons that function in complex loading environments and are subjected to multi-axial tensile, compressive and shear stresses, such as the supraspinatus tendon of the rotator cuff [7].

The supraspinatus tendon of the rotator cuff is subjected to such forces due to its role in shoulder movement and stability. The native collagen alignment of the tissue varies along the length of a tendon, with a more disorganized fiber matrix located at the tendon-to-bone insertion [8]. Previous studies have described the insertion region as a region of high stress [9]. As a result, tears and tendinopathies at the insertion region are common and often debilitating [10]. We have investigated the location-dependent response of collagen fibrils to mechanical loading in the mouse supraspinatus tendon. Using atomic force microscopy, we demonstrated that fibril stretch and sliding differ between the insertion and midsubstance regions under strain [11]. The insertion region exhibited greater fibril strain initially, followed by a decrease at higher tissue strains, while the midsubstance showed a more gradual response. These differences in fibril behavior underscore the influence of regional tendon organization on its mechanical response. However, a comprehensive multiscale analysis is warranted to elucidate further site-specific differences in structure, function, and gene expression.

Therefore, the objective of this study was to investigate regional differences in hierarchical structure, function, and gene expression of the murine supraspinatus tendon. We hypothesized that the insertion and midsubstance regions would demonstrate distinct structure, function, and gene expression. By utilizing mechanical testing, histology, transmission electron microscopy (TEM), and quantitative PCR (qPCR), we aimed to provide a detailed multiscale analysis of these regions. Our findings enhance the understanding of site-specific structure, function, and gene expression changes that may contribute to tendon pathology in the supraspinatus tendon.

## Methods

### Animal use & study design

This study was approved by the University of Pennsylvania Institutional Animal Care and Use Committee (protocol 806203) and carried out in strict accordance with guidelines established in the Guide for the Care and Use of Laboratory Animals of the National Institutes of Health (Bethesda, MD, USA). Wild-type C57BL/6 mice were housed in an AALAC accredited facility that maintained 12-hour light/dark cycles, temperatures between 20-26°C, and humidity between 30-70%. At Day 150, mice were euthanized with controlled flow-rate carbon dioxide. Supraspinatus tendons were evaluated for site-specific mechanics (n = 20/group), collagen fiber realignment (n = 20/group), collagen fibril morphology (n = 20/group), cellularity and cell shape (n = 7/group), and gene expression (n = 19/group).

### Biomechanical testing

Mice designated for biomechanical testing were frozen at -20°C until the day of testing. Mice were thawed at room temperature and the supraspinatus tendon-humerus complex from the left forelimb of each mouse was carefully dissected to remove extraneous tissue. Stain lines were applied for optical tracking at 0mm, 1mm, 2mm, and 2.5mm from the humeral insertion, where the insertion region (INS) was defined as the region between 0 and 1mm from the humeral insertion, and the midsubstance region (MID) was between 1 and 2mm from the humeral insertion [12]. A custom laser device composed of linear variable differential transformers (LVDTs), a CCD laser, and translation stages [13] was used to measure cross-sectional area of the supraspinatus tendon. Two sandpaper tabs were placed at the 2.5mm stain line and adhered with cyanoacrylate glue to prevent slippage. The humerus was secured in a custom construct with polymethyl methacrylate, and the construct was mounted on a material testing machine (Instron 5848) using a 10N load cell. All testing was conducted in a phosphate buffered saline bath at 37°C. Each sample was first preloaded to 0.025N. The testing protocol consisted of 10 cycles of preconditioning between 0.5-1.5% grip strain at 0.25 Hz, followed by a quasistatic ramp-to-failure at a strain rate of 0.1%/s. Images were captured during the quasistatic ramp-to-failure and used to optically track the insertion and midsubstance regions. Optical strain was used to calculate insertion and midsubstance moduli using a custom MATLAB (Natick, MA, USA) script [14]. Briefly, after manually selecting the insertion and midsubstance regions, deformable image registration was used to directly estimate the insertion and midsubstance deformation tensors for each frame and the Lagrangian strains of these regions were calculated using these deformation tensors. During the ramp-to-failure, collagen fiber realignment was quantified using cross-polarization imaging, and quantified regional fiber realignment data, represented as circular variance, were collected during the ramp to failure, normalized to the first discrete data point at 0% strain, and interpolated in MATLAB with a polynomial fit as a function of strain from the load-displacement data, as described [15].

### Assessment of cellularity & cell shape

For quantification of cellularity and cell shape, whole shoulders were grossly harvested and immediately fixed, decalcified, and processed for paraffin embedding using standard histological techniques. Briefly, coronal sections were cut to 7 µm thickness and stained with hematoxylin and eosin (H&E). The tendon proper, and not the surrounding sheaths, of the insertion and midsubstance regions of each sample were then imaged at 20x (S1 Fig) and evaluated using ImageJ for cellularity and cell shape, as described [16]. Briefly, cellularity was quantified as the cell density per area of region analyzed and cell shape was measured as the average aspect ratio of the nuclei of the region on a scale from 0 to 1, where 1 represented a perfect circular shape.

### Evaluation of collagen fibril morphology

Tendons assigned for fibril morphology analysis using TEM were prepared as described [16,17]. Following euthanasia, supraspinatus tendons were fixed in Karnovsky’s fixative (4% paraformaldehyde, 2.5% glutaraldehyde, 0.1M sodium cacodylate, 8.0mM calcium chloride), post-fixed and stained with 1% osmium tetroxide, dehydrated in ethanol, and embedded in Epon resin. Prior to embedding, tendons were separated into the insertion and midsubstance regions for analysis [16]. Tendons were then sectioned in the transverse plane, and ultrathin sections (60-80nm) were stained with UranyLess (EMS 22409) and 1% phosphotungstic acid. Images of the insertion and midsubstance regions (S2 Fig) were acquired at 60,000x magnification using a transmission electron microscope (JEOL 1010). For each sample, ten representative digital images were selected in a randomized order. Fibril diameters were measured in MATLAB using a custom code.

### Gene expression analysis

Following euthanasia, supraspinatus tendons were dissected, flash frozen in liquid nitrogen, and stored at -80°C. Tendons were later defrosted in RNAlater^TM^-ICE (ThermoFisher, AM7030), carefully separated into their insertion and midsubstance regions using a custom fixture, transferred to a phenol-based lysis reagent (QIAzol, Qiagen 79306), and mechanically homogenized using a pestle until the tissue dissolved. RNA was then isolated (Direct-zol RNA Microprep, Zymo, R2062), converted to cDNA (High-Capacity cDNA Reverse Transcription Kit, Applied Biosystems, 4368814), and preamplified for 15 cycles using 48 pre-selected Taqman primers for gene expression analysis. Preamplified cDNA was loaded into a Standard BioTools 48.48 Dynamic Array IFC (BMK-M-48.48, Standard BioTools, San Francisco, CA) and run with the 48 selected gene panel primers. Target genes included those of collagens, non-collagenous matrix, remodeling, growth factors, and cell markers (S1 Table). Cycle threshold (Ct) values were evaluated for each gene, including for the housekeeper genes *Abl1* and *Rps17*. DeltaCT (ΔCt) values were computed by subtracting gene Ct values from the average housekeeper Ct value for that sample.

### Statistics

Data points considered statistical outliers, identified as values falling below 1.5 times the interquartile range from the first quartile or above 1.5 times the interquartile range from the third quartile, were excluded from analysis. Data distributions were tested for normality using Shapiro-Wilk tests. For regional modulus, the comparison between insertion and midsubstance was made using a paired t-test. For regional collagen fiber realignment, comparisons between insertion and midsubstance were made using paired t-tests at each strain level. For regional fibril diameter distribution analysis, histograms were generated, and the insertion and midsubstance region distributions were statistically compared using Kolmogorov-Smirnov tests. Regional cellularity and cell shape data were compared between the insertion and midsubstance using a paired t-test. Principal component analysis (PCA) of insertion and midsubstance region gene expression data was performed via R. For analysis of individual gene expression, paired t-tests were conducted on ΔCt values between the insertion and midsubstance regions. Significance was set at p ≤ 0.05.

## Results

### Tendon mechanics and collagen fiber realignment

**Fig 1:**
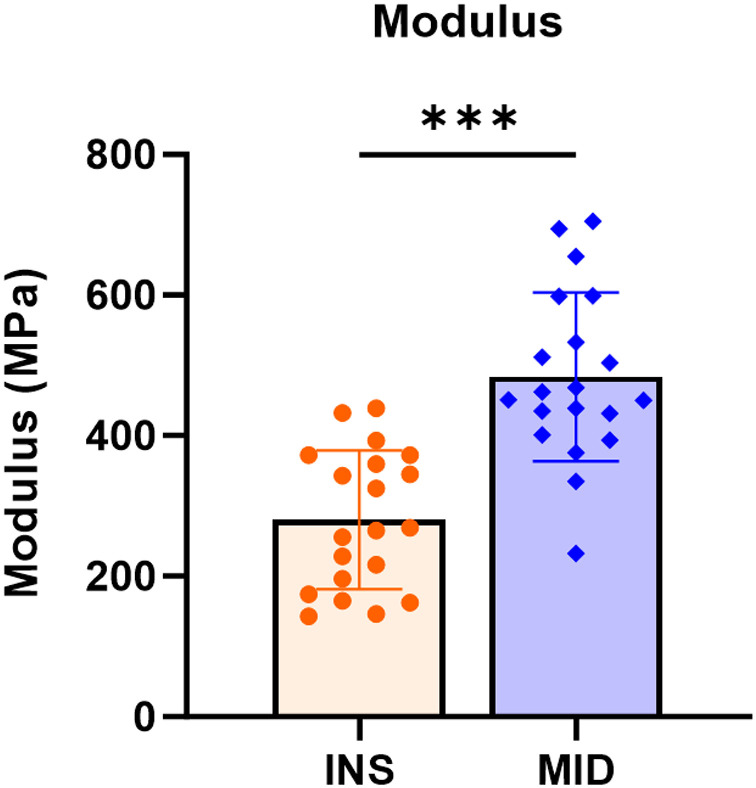
Site-specific differences in supraspinatus tendon modulus. Midsubstance regions exhibit a greater modulus compared to insertion regions. Data presented as mean ± standard deviation (***p ≤ 0.001).

**Fig 2:**
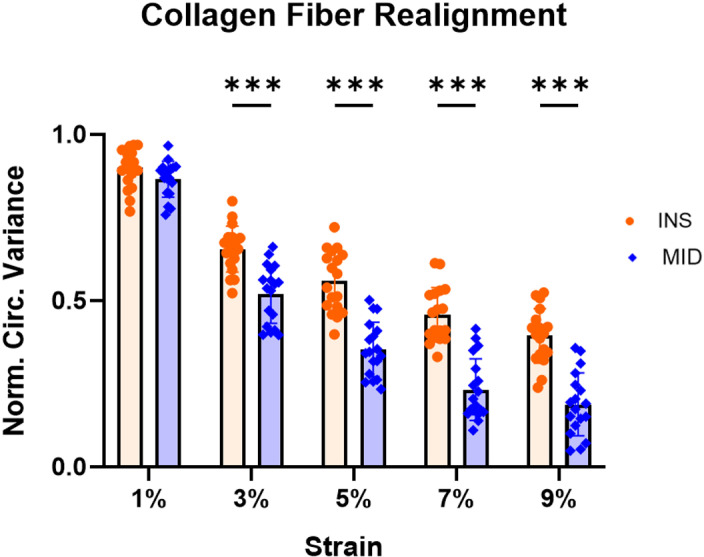
Site-specific collagen fiber realignment. Midsubstance regions demonstrated greater collagen fiber realignment (lower normalized circular variance) at strain values between 3 and 9%. Circular variance data were collected during the ramp to failure and normalized to the first discrete data point at 0% strain. Data presented as mean ± standard deviation (***p ≤ 0.001).

Supraspinatus tendons demonstrated greater modulus in the midsubstance region relative to the insertion region (Fig 1). Additionally, the midsubstance region demonstrated increased collagen fiber realignment compared to insertion region, as evident by lower normalized circular variance values from 3-9% strain, encompassing the toe and linear elastic regions of these tendons (Fig 2).

### Tendon cellularity & cell morphology

**Fig 3:**
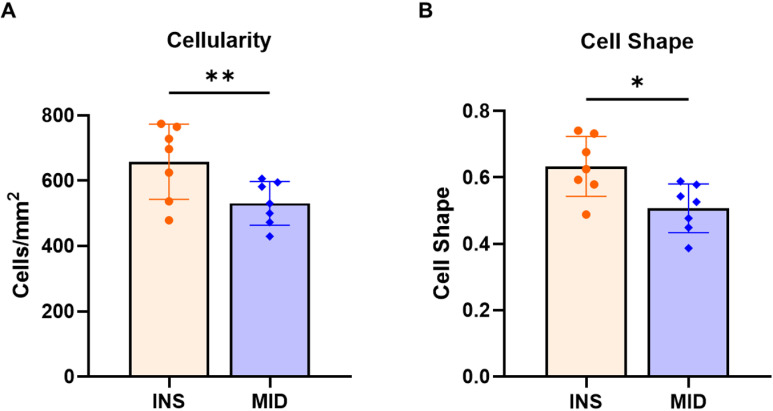
Site-specific cellularity and cell shape. Insertion regions demonstrated greater A) cellularity and greater B) cell shape, indicating more rounded cells, compared with midsubstance regions. Data presented as median ± interquartile range (*p ≤ 0.05, **p ≤ 0.01).

Quantitative evaluation of the insertion and midsubstance regions resulted in a greater cellularity (Fig 3A), and a higher cell shape score (Fig 3B), indicating more rounded cells, in the insertion region relative to the midsubstance region.

### Collagen fibril morphology

**Fig 4:**
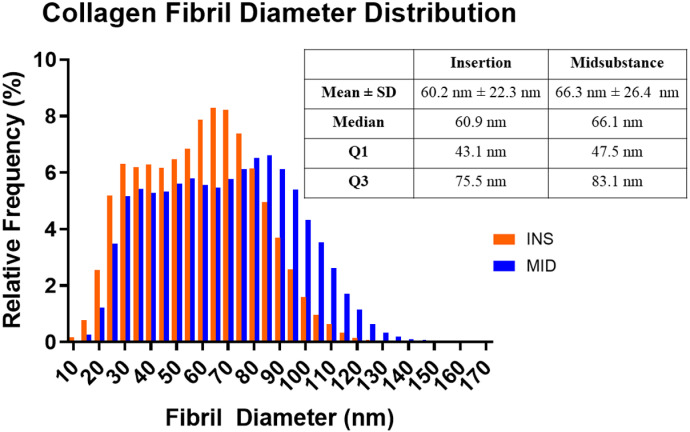
Site-specific collagen fibril diameter distributions. Fibril diameter distributions demonstrate a shift towards smaller diameter fibrils in the insertion region compared with midsubstance region.

Collagen fibril diameters were analyzed using transmission electron microscopy in the insertion and midsubstance regions. Fibrils in the insertion and midsubstance regions exhibited normal circular cross-sectional profiles throughout the tendon extracellular matrix. The midsubstance region exhibited a distribution of collagen fibril diameters ranging from 10.4 nm – 167.8 nm, with a mean ± standard deviation of 66.3 nm ± 26.4 nm, and a median (interquartile range) of 66.1 nm (35.6 nm). At the insertion region, there was a shift toward smaller diameter fibrils compared to the midsubstance with diameters ranging from 9.6 nm – 140.5 nm, a mean ± standard deviation of 60.2 nm ± 22.3 nm, and a median (interquartile range) of 60.9 nm (32.4 nm) (Fig 4).

### Gene expression

**Fig 5:**
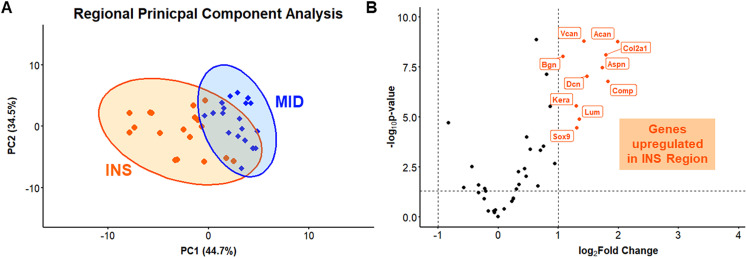
Site-specific principal component analysis and volcano plot. A) PCA demonstrated clustering based on region. B) Volcano plot shows differential gene expression with upregulated genes expressed in the insertion region relative to the midsubstance region.

**Fig 6:**
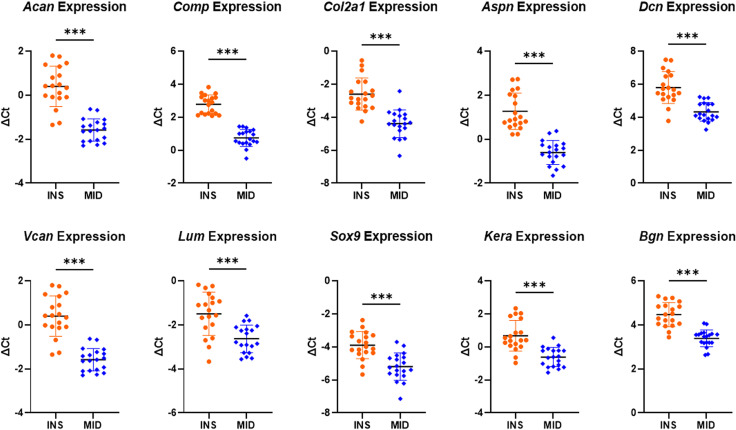
Site-specific gene expression plots. ΔCt gene expression data from upregulated genes in the insertion region relative to the midsubstance region. Data presented as mean ± standard deviation (***p ≤ 0.001).

Principal component analysis revealed clustering by region with the first principal component (PC1) accounting for 44.7% of the variance while principal component 2 (PC2) accounts for 34.5% of the variance for a total of 79.2% between them (Fig 5A). Specifically, *Acan*, *Comp*, *Col2a1*, *Aspn*, *Dcn*, *Vcan*, *Lum*, *Sox9*, *Kera* and *Bgn* were upregulated in the insertion region relative to the midsubstance region (Figs 5B and 6).

## Discussion

The objective of this study was to investigate site-specific structure, function, and gene expression differences in the mature murine supraspinatus tendon. Consistent with our hypothesis, we demonstrated regional differences in modulus, collagen fiber realignment, cellularity and cell shape, collagen fibril diameter distributions, and gene expression. Mechanical testing revealed that the midsubstance region demonstrated a greater modulus and greater collagen fiber realignment compared to the insertion region. Histological scoring demonstrated greater cellularity and more rounded cells in the insertion region. TEM analysis revealed a shift towards smaller diameter fibrils in the insertion region relative to the midsubstance region. Gene expression analysis further revealed region-specific differences, with several proteoglycans and chondrogenic markers upregulated in the insertion region relative to the midsubstance.

The regional differences observed in mechanical properties and collagen fiber realignment suggest that different regions may undergo distinct loading conditions *in vivo*, leading to localized remodeling and resulting in varied fiber network configurations, including changes in collagen fiber alignment, cross-links, and fiber-fiber and fiber-matrix interactions. Our findings of reduced modulus and collagen fiber realignment in the insertion regions are supported by previous mouse supraspinatus tendon data which demonstrated reduced insertion modulus and reduced insertion collagen fiber realignment in the transition and linear regions of these tendons [18]. Furthermore, the tendon-to-bone insertion region of the supraspinatus tendon experiences higher strains and has been shown to have a more disorganized fiber distribution compared to the tendon midsubstance [19–21]. These potential collagen microstructural changes across different locations may influence their ability to respond to load in a coordinated or consistent manner. The lower moduli and higher normalized circular variance values (indicating decreased reorganization) observed at the insertion region compared to the midsubstance location suggest that the multiaxial loads at the supraspinatus tendon insertion could impact both the tissue’s structure and mechanics. This may be due to the insertion region being the primary source of force transfer from bone to tendon and a relatively weaker region of tendon mechanically, making it more susceptible to mechanical changes [22].

The differences observed in cellularity and cell shape between the insertion and midsubstance regions highlight regional adaptations and cell heterogeneity of the supraspinatus tendon in response to different mechanical environments. Our finding of increased cellularity in the insertion region is supported by previous studies, where tendons subjected to complex loading conditions, particularly in the tendon-to-bone insertion regions, have shown increased cellularity due to heightened remodeling activity required to manage the multiaxial forces present at these sites [9,19]. Tendon insertions typically experience compressive loads, which induce fibrocartilage formation, increased proteoglycan content, and higher cell densities [7,23]. The distinct cell shape observed in the insertion region, characterized by more rounded cells, further underscores the site-specific heterogeneity of cell types leading to site-specific functional specialization. This rounded morphology, indicative of greater presence of fibrochondrocytes, can be attributed to the multiaxial loading conditions such as compression and shear, which contrast the primarily unidirectional tensile forces to which the midsubstance is exposed [6,20]. Previous studies have shown that tenocytes in regions subjected to tensile forces, such as the tendon midsubstance, maintain a more elongated or spindle-shaped morphology, which is optimized for tensile strength [4,24]. Our study supports these findings since we observed reduced cell shape and increased modulus in the midsubstance region. The site-specific differences in cellularity and cell shape are indicative of localized mechanobiological responses, where mechanical cues from the environment directly influence cellular behavior and tissue structure-function. The higher cellularity in the insertion region may also reflect an increased capacity for tissue repair and remodeling, which is critical for managing the higher mechanical demands at the tendon-bone interface [22,25].

The distribution of fibril diameters was shifted towards smaller diameters at the insertion region compared to the midsubstance. Previous studies have shown that fibril diameter distributions can differ from the bone-tendon junction to the myotendinous junction in superficial digital flexor tendons [26,27]. Among extracellular matrix structures, collagen fibrils play a role in transmitting tensile forces, as supported by many reports that the *in vivo* mechanical properties of tendons and ligaments are related to collagen fibril diameter and arrangement [e.g., [28–30]]. Collagen fibrils opposing unidirectional tensile forces, as in the midsubstance region, have demonstrated larger diameters, compared to relatively smaller diameter collagen fibrils resisting multidirectional forces, as in the insertion region, skin, and blood vessel walls [31–34].

Our results suggest potential multiscale structure-function mechanisms relating macroscale tissue mechanical behavior to microscale collagen fiber realignment and nanoscale fibril morphology [35]. Specifically, in the insertion region, decreased collagen fiber realignment, indicative of an altered site-specific structural response to load, in conjunction with smaller diameter fibrils unable to withstand the same loading magnitude results in inferior mechanical properties relative to the midsubstance region. This induces earlier accumulation of damage at the fiber and fibril levels which propagates to the macroscale, ultimately leading to premature tendon failure at the tendon insertion, as observed in all supraspinatus tendon samples.

The upregulation of genes such as *Acan*, *Comp*, *Col2a1*, *Sox9*, *Aspn*, *Dcn*, *Vcan*, *Lum*, *Kera*, and *Bgn* in the tendon insertion region highlights the functional specialization of this region, which is subjected to compressive and multiaxial forces. Greater expression of *Col2a1* and *Sox9* in the insertion region indicates greater quantity of fibrochondrocytes in the insertion region. This adaptation towards fibrocartilage formation, a tissue better suited to resist compressive loading [7,36–38] was further supported by our histological finding of more rounded cells in this region relative to the more elongated tenocytes observed in the midsubstance region. Fibrocartilage is essential at insertion regions because of the need to handle both tensile and compressive stresses during load transmission from tendon to bone. The upregulation of *Vcan* and *Comp* further supports the presence of fibrocartilaginous tissue and plays critical roles in tissue hydration and compressive resistance [39–42]. Furthermore, the upregulation of *Acan* in the insertion region aligns with its function in fibrocartilaginous tissues and provides insight into how tendons adapt to compressive loads at the tendon-bone interface. *Acan* helps retain water in the tissue due to its glycosaminoglycan (GAG) side chains, contributing to the ability of the tendon insertion region to withstand compressive forces and maintaining tissue integrity in regions subjected to multiaxial forces, thus complementing the actions of *Vcan* and *Comp* [43–46].

Class I small leucine-rich proteoglycans (SLRPs), *Dcn*, *Bgn*, and *Aspn*, and class II SLRPs, *Lum* and *Kera*, are and were upregulated in the insertion region relative to the midsubstance. Previous studies [12,47–52] have demonstrated these class I SLRPs are integral for maintaining collagen fibril structure, fiber realignment, and mechanical properties of mature and aged tendon. Since we have demonstrated that changes in collagen fibril structure, fiber realignment, and mechanical properties after loss of *Dcn* and *Bgn* differs from one tendon to another [47], it is not surprising that these structural and functional properties may also be altered from the insertion region to the midsubstance along a single tendon. Recent studies analyzing the size of dermatan sulfate filaments found location-dependent length and orientation of filaments between collagen fibrils in horse superficial digital flexor tendon [52], providing additional evidence for location-dependent functions of these SLRPs. Additionally, class II SLRPs *Lum* and *Kera* play an important role in regulating collagen fibrillogenesis, morphology and mechanical function, suggesting that their upregulation at the insertion region may have contributed to differences observed in overall structure and function [53–56].

While our study provides valuable insights into the multiscale structure-function relationships in murine supraspinatus tendons, it is not without limitations. We investigated the supraspinatus tendon to expand upon regional differences that we observed at the fibril level [11] and because tears and tendinopathies occur more frequently at its insertion region [10]. However, site-specific changes in structure, function and gene expression may differ between tendons with various mechanical requirements. Another limitation is the lack of matrix compositional analysis. Although we observed site-specific differences in expression of key matrix genes, it is unknown if these gene expression changes correspond to compositional protein-level changes in either region of the tendon. Future studies can elucidate these relationships by implementing proteomic analyses, such as nano-flow liquid chromatography tandem mass spectrometry (LC-MS/MS), to identify site-specific differences in protein expression [57]. Furthermore, future studies may benefit from implementation of finite element analysis (FEA) methods to model how differences in the local mechanical environment of the tendon correspond with the site-specific differences observed in mechanical function and collagen fiber realignment. Defining these relationships may provide further insight into understanding extrinsic mechanisms contributing to the site-specific differences observed in this study. Lastly, this study only investigated regional structure, function, and gene expression changes in mature day 150 mice. Future studies could benefit from exploring the effects of aging on regional structure and function in supraspinatus tendons, as the incidence of rotator cuff tears and tendinopathies increases with age and is a common clinical problem in the geriatric population [58].

This study demonstrates regional variations in mechanical properties, collagen fiber realignment, cellularity and cell shape, fibril morphology, and gene expression of the mouse supraspinatus tendon. These findings highlight the importance of considering tendon heterogeneity when evaluating tendon structure, function, and gene expression and underscore the complex, adaptive nature of tendons and suggest that each region is uniquely tailored to meet its specific mechanical demands. Additionally, the site-specific structural, functional, and gene expression changes observed in this study may inform future studies aiming to understand regional tendon pathology (i.e., tears predominantly occur at the insertion region of the supraspinatus tendon). Taken together, these findings provide a comprehensive understanding of the site-specific hierarchical structure, function, and molecular characteristics of the supraspinatus tendon, highlighting its complex regional heterogeneity.

## Supporting Information

Fig S1 Representative H&E images of the insertion and midsubstance regions. (TIF)

Fig S2 Representative TEM images of the insertion and midsubstance regions. (TIF)

Table S1 Panel of Target Genes. (XLSX)
