# Supplementary figures and images for: Mature murine supraspinatus tendons demonstrate regional differences in multiscale structure, function and gene expression
Source: PLoS One. 2025 Feb 20;20(2):e0318809. doi: 10.1371/journal.pone.0318809 (PMC11841869; doi:10.1371/journal.pone.0318809)

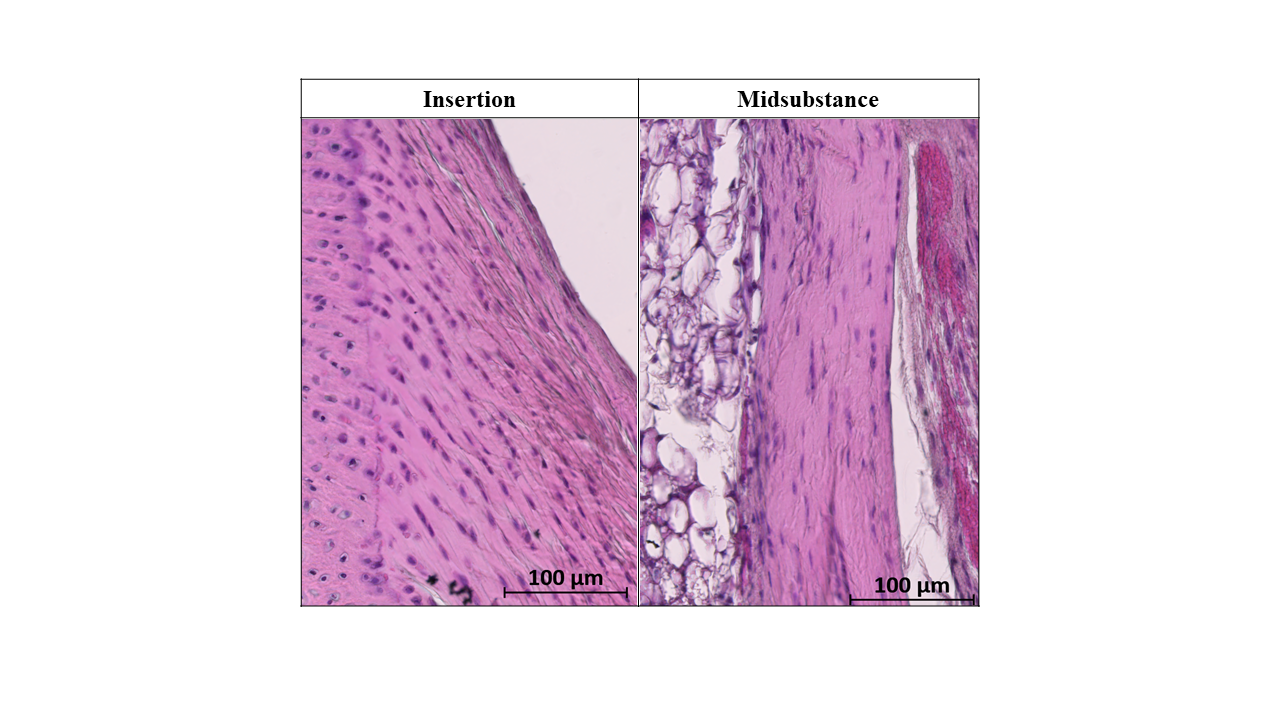

Supplement: Fig S1 — (TIF) [file pone.0318809.s001.tif]

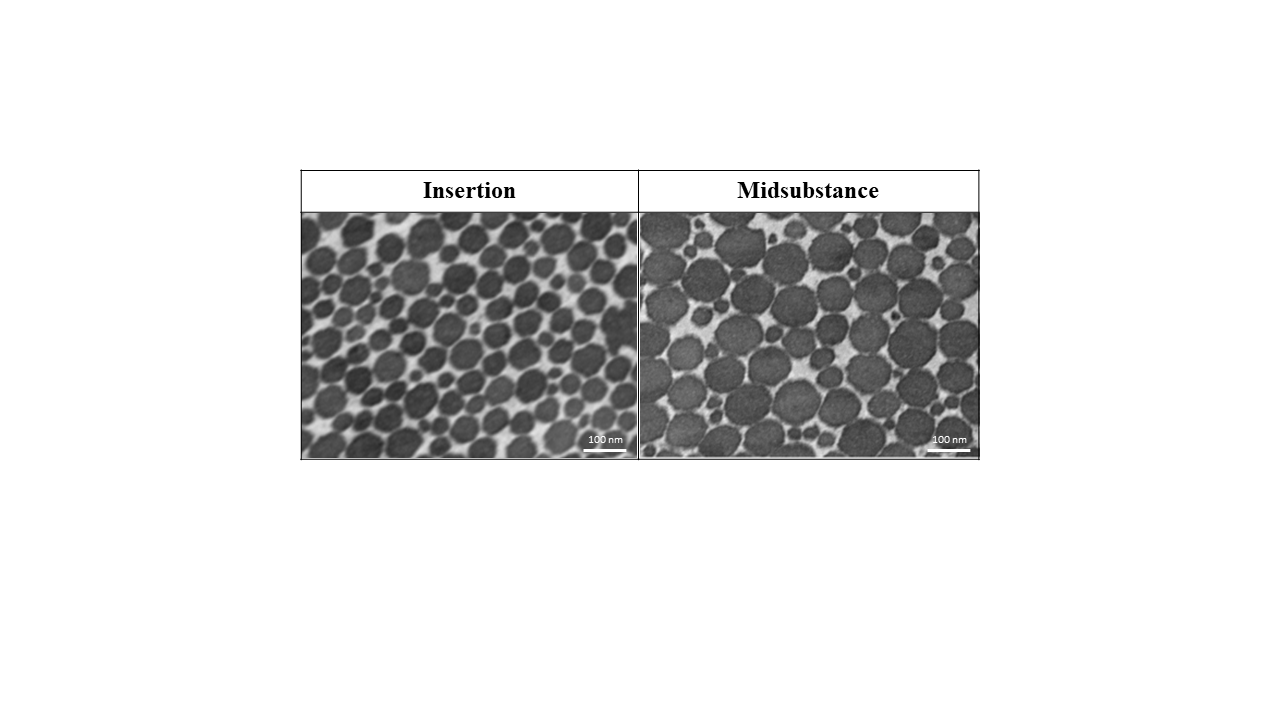

Supplement: Fig S2 — (TIF) [file pone.0318809.s002.tif]
